# A Fatigue Model to Predict Interlaminar Damage of FRP Composite Laminates Subjected to Mode I Load

**DOI:** 10.3390/polym15030527

**Published:** 2023-01-19

**Authors:** Safdar Ali Khan, Seyed Saeid Rahimian Koloor, Wong King Jye, Geralt Siebert, Mohd Nasir Tamin

**Affiliations:** 1 Faculty of Mechanical Engineering, Universiti Teknologi Malaysia, 81310 Johor Bahru, Johor, Malaysia; 2 Institute for Structural Engineering, Department of Civil Engineering and Environmental Sciences, Universität der Bundeswehr München, Werner-Heisenberg-Weg 39, 85577 Neubiberg, Munich, Germany

**Keywords:** cyclic cohesive zone model, damage mechanics, interface fatigue crack, interlaminar property degradation, fiber-reinforced polymer composite laminates

## Abstract

In fiber-reinforced polymer (FRP) composite laminate structures operating under fluctuating stresses, interface delamination is seen as one of the significant damage mechanisms. The constant degradation of their relatively low interlaminar strength and stiffness are the primary reasons for delamination. This study develops an interlaminar fatigue damage model to quantify the mechanics of the damage process and address the reliability of composite structures. The model considers the failure process in two stages: (1) damage due to degradation of interlaminar elastic properties, and (2) damage due to dissipation of fracture energy through the damage evolution process. The model is examined for a case study of mode I fatigue loading of a carbon-fiber-reinforced polymer (CFRP) composite laminate. The results show that the interlaminar normal stress is confined to the crack front region, with tensile stress peaks at 70% of the interlaminar strength. Furthermore, a stable interface crack growth is predicted initially, followed by a sudden crack “jump” at 14,000 cycles. The simulation results are compared with the experimental data, with very good agreement, showing a successful validation of the fatigue model.

## 1. Introduction

Fiber-reinforced polymer (FRP) composite laminates have attracted attention for many industrial applications in recent decades. Their high strength-to-weight ratio makes them desirable for aerospace applications. Typical applications include the fuselage and spoilers of aircraft, the skin of wind turbine blades, and vehicle components. These composite structures undergo static and fatigue loading throughout their service life. Under such loading, various types of inter- and intralaminar fatigue damage are predominantly caused by cyclic loading, especially due to the low strength and toughness of the different constituents of laminated composites, affecting the reliability of FRP composite structures [1,2]. As a result, the development of fatigue damage models is valuable as a tool for complete insight into the mechanics of deformation and failure of the material, as well as for the reliability assessment of composite structures. 

Composite structures as a part of vehicles’ bodies are often designed to bear complex loads that appear in the form of lateral loads, bending, twisting, etc. [3,4]. Such loading causes various models of failure in laminated composites, including matrix yielding and cracking, fiber/matrix interface debonding, fiber pullout, fiber buckling, fracture, and interface delamination [5,6,7]. Multiple interface delamination is among the most frequently seen failure modes, often occurring under mixed-mode loading conditions. The constitutive damage models of interface failure are derived to consider damage modes I and II, along with their interaction; therefore, the knowledge of the mechanics and mechanisms of interface damage in every single mode is a necessary requirement to build the constitutive models [8,9]. In this regard, this work is dedicated to investigating the mode I interface damage to FRP composite laminates.

The different combinations of failure modes in FRP composite laminates are observed due to the relatively high amplitude and mean stress components of the operating loads. The growth behavior of near-threshold interface fatigue cracks in the carbon-fiber-reinforced polymer (CFRP) composite laminates is dominated by matrix cracking and interface delamination under mode I crack loading [10,11]. Because of the low interlaminar toughness and strength, interface delamination is the most common failure mechanism [12,13]. Interface delamination has also been observed because of matrix cracking in the adjoining lamina [14,15]. The occurrence of interface delamination has been shown to result in significant material stiffness degradation [16,17]. These findings indicate that the composite structural components are extremely vulnerable to failure due to interface delamination. Again, accurate interlaminar fatigue damage and failure models are required for assessing the structural reliability of FRP composite laminates.

The cohesive zone model (CZM) was used to simulate interface delamination in FRP composite laminates under static loading conditions [18]. For the CZM, various softening laws have been investigated [19,20]. The difficulties in quantifying interface properties and model parameters have been tackled sufficiently. These include standard test methods [21,22] and a combined experimental–FE approach [23]. The CZM has also been incorporated into finite element analysis (FEA) software [24] and used in FE simulations of FRP composite laminates’ interface failure process [25,26,27,28]. 

The interface fatigue failure process has previously been investigated using both experimental and numerical techniques. Interlaminar fatigue models of various types have been developed using continuum stress-life, fracture mechanics, and cohesive damage approaches. The linear elastic fracture mechanics (LEFM) approach uses the exponential growth law to describe the interlaminar fatigue crack growth response, which is expressed in terms of strain energy release rates [29]. The damage-based cohesive models were developed to describe the onset of interlaminar fatigue damage and its progression to interface delamination. The cohesive damage models have the additional capability of smoothing the stress singularities at the crack tip when compared to the fracture mechanics approaches. Additionally, nonlinearities in material and geometry are conveniently implemented [30]. Moura et al. (2014) and Moroni et al. (2011) used the CZM to simulate interface fatigue in mode I and mixed modes for adhesive joints [31,32]. Turon et al. (2007) investigated mesh size effects in CZM simulations for the cohesive layer [33].

Given the CZM’s success in modeling the fracture phenomena of quasi-brittle materials, the CZM’s capability could be extended to the simulation of fatigue crack growth. Allegri [34] proposed a semi-analytical CCZM that reveals interactions between the material properties describing quasi-static tearing, fatigue life, and crack propagation rates. Incorporating a fatigue damage property into the constitutive damage model is essential for developing a cyclic CZM [35]. Yang et al. [36] first projected a cyclic CZM for fatigue crack growth in materials that exhibit quasi-brittle behavior, using a reduction in elastic modulus for the unloading back to the origin. The accumulation of fatigue damage during loading–unloading cycles below the quasi-static envelope was made possible by stiffness degradation, allowing the simulation of fatigue crack growth. In addition, Roth et al. [37,38] proposed a cyclic CZM and the concept of cohesive zone potential to physically measure stiffness degradation. However, because fatigue damage was accumulated after the first cycle (in these cyclic CZMs based on stiffness degradation), crack initiation was not explicitly considered [39]. Khoramishad et al. [40,41,42] hypothesized that fatigue-induced strength degradation would predominate in fatigue crack growth and created a CCZM based on strength degradation. However, the aforementioned cyclic CZMs are all adopted to the condition that the unloading path returns to zero deformation, which is not accurate in the low-cycle fatigue content of quasi-brittle materials. Parrinello and Benedetti [43] proposed elastic–plastic cohesion laws for polycrystalline low-cycle fatigue, taking the cohesive–frictional behavior into account [44].

In this study, a new cyclic cohesive zone model (CCZM) was developed and employed for the reliability prediction of FRP composite laminates under mode I crack loading conditions. The theoretical damage model of the interface was developed by combining two models, i.e., (1) a property degradation model, and (2) a fatigue life model. The models encompass the interface’s typical fatigue life and recognize the fatigue degradation of interlaminar strength and stiffness properties during the damage progression to the onset of crack nucleation. The dissipation of fracture energy governs the separation of the material point in the interface. The model was embedded in a finite element analysis (FEA) package using the subroutine UMAT and used to investigate the failure and damage during mode I fatigue loading of CFRP composite laminates. A comparison of the model’s FE-predicted results and experimental data (from the literature) showed a successful validation of the model. 

## 2. Cohesive Zone Model for Static Loading

**Figure 1:**
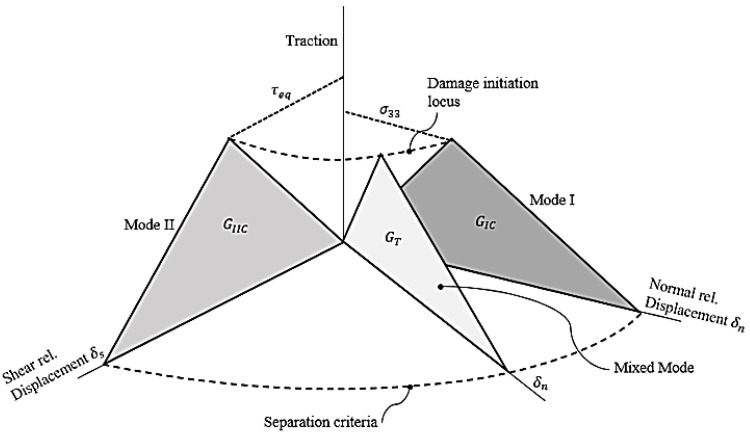
Bilinear traction–separation softening law for the cohesive interface that faces quasi-static mixed-mode loading.

The interfaces between two laminas in an FRP composite laminate are assumed to have cohesive behavior. In addition, the interface is relatively thin, such that only the out-of-plane normal and in-plane shear stress components act on the mathematically “zero-thickness” layer. Bilinear traction–displacement softening law is assumed for the cohesive interface under the quasi-static loading, as schematically shown in Figure 1. This cohesive zone model (CZM), describing the criterion for the onset of interlaminar damage, is given as follows [18]:

(1) $$\sqrt{{\left(\frac{\langle \sigma_{33}\rangle }{T_{0}}\right)}^{2}+{\left(\frac{\tau_{13}}{S_{0}}\right)}^{2}+{\left(\frac{\tau_{23}}{S_{0}}\right)}^{2}}=1;\langle \sigma_{33}\rangle =\{\begin{array}{c} 0\;\;\;\;;\;\sigma_{33}\leq 0\\ \sigma_{33}\;;\;\sigma_{33}>0 \end{array}$$

where $\sigma_{33}$ is the tensile normal stress and ($\tau_{31},\tau_{32}$) are the components of shear stress on the interface constituent. The interlaminar normal tensile and shear strength are represented by *T*_0_ and *S*_0_*,* respectively. The normal stress as a result of compressive load, is not affecting the interlaminar damage process. In addition, the shear stress terms diminish under the mode I crack loading. The subsequent damage evolution process causing the separation of the interface material point is governed by the dissipation of interlaminar strain energy. The fracture energy under individual mode I is given by the area below the traction–relative displacement curve, as follows:

(2) $$G_{IC}=\frac{1}{2}T_{0}\delta_{f}^{n}$$

where $T_{0}$ is the interface normal strength and $\delta_{f}^{n}$ is the relative displacement of the interface at fracture in mode I. Similarly, for isolated mode II, the fracture energy is given as follows:

(3) $$G_{IIC}=\frac{1}{2}S_{0}\delta_{f}^{s}$$

where *S*_0_ is the interface shear strength and $\delta_{f}^{S}$ is the relative displacement at fracture in mode II. The strain energy release rate $G_{T}$ under the mixed-mode (modes I and II) crack loading condition is described as follows [45]:

(4) $$G_{T}=G_{IC}+\left(G_{IIC}-G_{IC}\right){\left(\frac{G_{II}}{G_{I}+G_{II}}\right)}^{\eta }$$

where *G_IC_* and *G_IIC_* are critical strain energy release rates in mode I and mode II loading, respectively. The exponent *η* represents the degree of interaction of each loading mode, taken as 1.45. *G_II_* and *G_I_* represent the energy release rates, and their ratio in Equation (4) indicates the participation of individual modes. In the isolated mode I loading condition, $G_{II}=0\;$ and the equation is reduced to $G_{T}=G_{IC}$. In the isolated mode II crack loading conditions, $G_{I}=0$ and the equation is reduced to $G_{T}=G_{IIC}$. The critical material point on the interface separates when the total fracture energy is dissipated.

The CZM properties’ parameters were determined through the isolated mode testing of CFRP composite laminate specimens. At the damage initiation point, the local maximum stress attained is referred to as the interface strength. A validated hybrid FE–experimental approach was employed to extract the CZM parameters. The hybrid FE–experimental approach is detailed in the published literature [23]. Table 1 lists the resulting interface properties. 

## 3. Interlaminar Fatigue Damage Model

**Figure 2:**
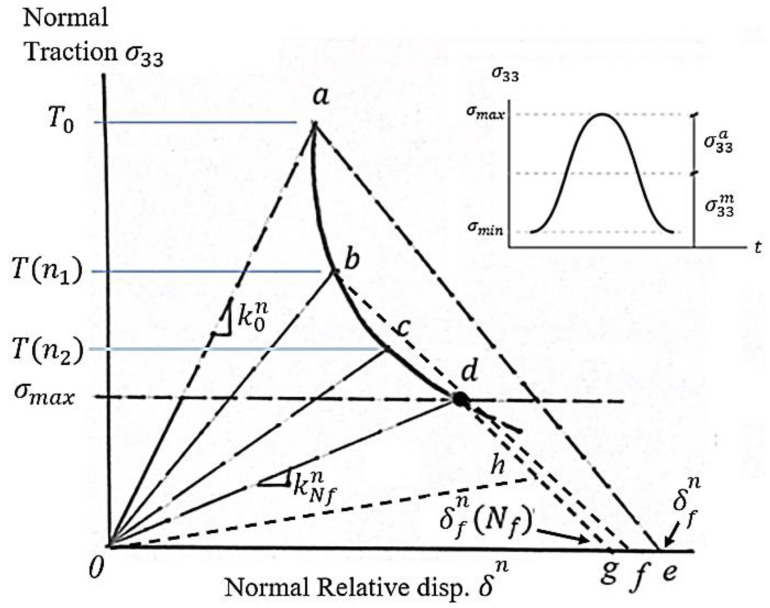
Interlaminar fatigue damage model illustrated for the mode I crack loading.

Interlaminar strength and stiffness degradation, the fatigue life of the interface, and characteristic damage evolution under fatigue loading conditions were employed to quantify the process of the mechanics of interlaminar fatigue damage. Figure 2 illustrates the mechanics of the interlaminar fatigue damage model through the evolution of normal traction–relative displacement curves at the critical interface material point experiencing fluctuating mode I crack loading. The interface was loaded with the maximum traction of $\sigma_{max}$ due to the application of constant-amplitude external loading with a load ratio (κ>0). The load ratio κ is the ratio of minimum to maximum stress. The interlaminar fatigue damage process—from pristine condition to full separation—of any material point of interface is composed of two stages: The first stage of fatigue damage is caused by the degradation of the interface properties due to fatigue loading. Curves *a-b-c-d* indicate this fatigue damage stage as the fatigue load cycles advance. The end of this curve, point *d*, represents the onset of the crack nucleation event. The stage of fatigue damage evolution follows the first step of damage. This second stage of damage is governed by fracture energy dissipation (Δ*0-d-g*) that starts at point *d* (nucleation onset) and ends at point *g* (separation of the material point). This implies that the governing damage mechanisms for both stages of damage are different and, therefore, should be treated separately. As soon as the material point on the interface separates, the local stress reduces to zero and the load is redistributed to nearby elements to maintain the forces’ equilibrium. All of the subsequent material points on the interface experience a similar fatigue damage process. These separated material points collectively create a fatigue crack that advances with the increasing number of load cycles until a final fracture of the whole interface. 

### 3.1. Cyclic Cohesive Zone Model for Mode I Crack Loading 

The hypothesis of the fatigue damage model is based on the gradual deterioration of the properties of the interface (i.e., normal strength $T_{0}$, fracture energy $G_{IC}^{0}$, and penalty stiffness $k_{0}^{n}$). Curve *0ae* is the reference bilinear softening response at the beginning of the load cycles or, alternatively, a quasi-static response. The interlaminar tensile strength $T_{0}$, penalty stiffness $k_{0}^{n}$, and fracture energy $G_{IC}^{0}$ degrade with the elapsed of the load cycles (*n_1_*), to new values of $T\left(n_{1}\right)$, $k^{n}\left(n_{1}\right)$, and $G_{IC}\left(n_{1}\right)$ signifying the accumulation of fatigue damage. The initial critical strain energy release rate $G_{IC}^{0}$ represented by the area under the Δ*0ae* is reduced to the area beneath the Δ*0bf.* Further fatigue load cycles (*n_2_*) would continue the degradation of these properties to $T\left(n_{2}\right)$, $k^{n}\left(n_{2}\right)$, and $G_{IC}\left(n_{2}\right)$, while the apex of the traction–relative displacement curve follows the path *a-b-c-d*. The interlaminar tensile strength could only degrade to the maximum stress level $\sigma_{max}$ of the load cycles (point *d*), where the interface crack nucleation process begins. The evolution of interlaminar fatigue damage due to property degradation $D_{f}$, which follows the path *a-b-c-d* from the pristine condition $(D_{f}=0$) to the onset of the crack nucleation ($D_{f}=1.0$), is given as follows:

(5) $$\sqrt{{\left(\frac{T_{0}-T\left(n\right)}{T_{0}-\sigma_{max}}\right)}^{2}+{\left(\frac{S_{0}-S\left(n\right)}{S_{0}-\tau_{max}}\right)}^{2}}\leq D_{f}$$

The variables T(n) and S(n) are the residual tensile and shear strength, respectively, corresponding to *n* elapsed load cycles. At this stage (point *d*), the damage due to fracture energy dissipation $D_{e}=0$. Following the onset of the interface crack nucleation process, the fatigue damage due to fracture energy dissipation $D_{e}$ under mode I crack loading is governed by the dissipation of the residual interlaminar strain energy release rate $G_{I}\left(n\right)$. Further fatigue load cycles would degrade the fracture energy to the path *o-h-g*. Upon further fatigue loading, the apex of the traction–relative displacement curve follows the line *h-g,* and all of the fracture energy is dissipated at point g *(*$D_{e}=1.0$*).* This marks the separation of the material point on the interface. The damage variable $D_{e}$ quantifies the fatigue damage process from the onset of crack nucleation (point *d*, $D_{e}$ = 0) to the final separation of the material point (point *g*, $D_{e}$ = 1.0), as follows:

(6) $$D_{e}=1-\frac{G_{I}\left(n\right)}{G_{IC}}$$

Separation of the critical interface material point occurs when $G_{I}\left(n\right)$ reduces to zero or $D_{e}=1.0.$ This signifies the nucleation of the interlaminar fatigue crack. Adjacent separated material points collectively form the structural interface crack. This enables the interface crack propagation process in the cohesive interface plane to be simulated.

### 3.2. Interlaminar Property Degradation Model

The interrupted fatigue tests under different applied stress conditions ($\kappa ,\;\sigma_{max}$) and the number of accumulated stress cycles *n* was used to establish the degradation of the interface properties. These properties include normal stiffness $k_{0}^{n}$, fracture energy $G_{IC}^{0}$, and tensile strength $T_{0}$ for the interface. The tests were carried out on CFRP composite laminates with DCB ENF specimen geometry under specific fatigue loading conditions ($\kappa ,\;\sigma_{max},\;n$). These pre-fatigued specimens were then loaded in the tensile testing machine for a quasi-static loading until fracture, and the load–displacement curves were documented. A validated hybrid FE–experimental technique was then employed for each test specimen to establish the normal penalty stiffnesses and interface strength. Experimental load and displacement were used for the calculation of fracture energy for the isolated loading mode. A hybrid FE–experimental technique has already been published [23] that guides the extraction of interface properties of FRP composite laminates under damage due to cyclic loads. It should be noted that throughout the interface’s fatigue life, the properties degrade in a similar manner. As a result, the degraded strength property data can be stated in their normalized form as follows [48]:

(7) $$\frac{T\left(n\right)-\sigma_{max}}{T_{0}-\sigma_{max}}={\left\{1-{\left[\frac{\log\left(n\right)-\log\left(0.5\right)}{\log\left(N_{f}\right)-\log\left(0.5\right)}\right]}^{\beta }\right\}}^{\frac{1}{\alpha }}\;$$

By having static strength $T_{0}$, maximum applied stress $\sigma_{max}$, the number of cycles to failure *N_f_* (related to the specific loading state), and curve fitting parameters α and β, the residual interface strength at any number of cycles T(n) can be calculated. By executing a similar process, the normalized residual penalty stiffness can be calculated as follows [48]:

(8) $$\frac{k^{n}\left(n\right)-\frac{\sigma_{max}}{\delta_{0}^{n}}}{k_{0}^{n}-\frac{\sigma_{max}}{\delta_{0}^{n}}}={\left\{1-{\left[\frac{\log\left(n\right)-\log\left(0.5\right)}{\log\left(N_{f}\right)-\log\left(0.5\right)}\right]}^{\lambda }\right\}}^{\frac{1}{\gamma }}\;$$

where $k^{n}\left(n\right)$ is residual interface stiffness, $k_{0}^{n}$ is interface stiffness for quasi-static cases, $\delta_{0}^{n}$ is the relative displacement of the interface at damage onset, and γ and λ are curve-fitting parameters. 

**Figure 3:**
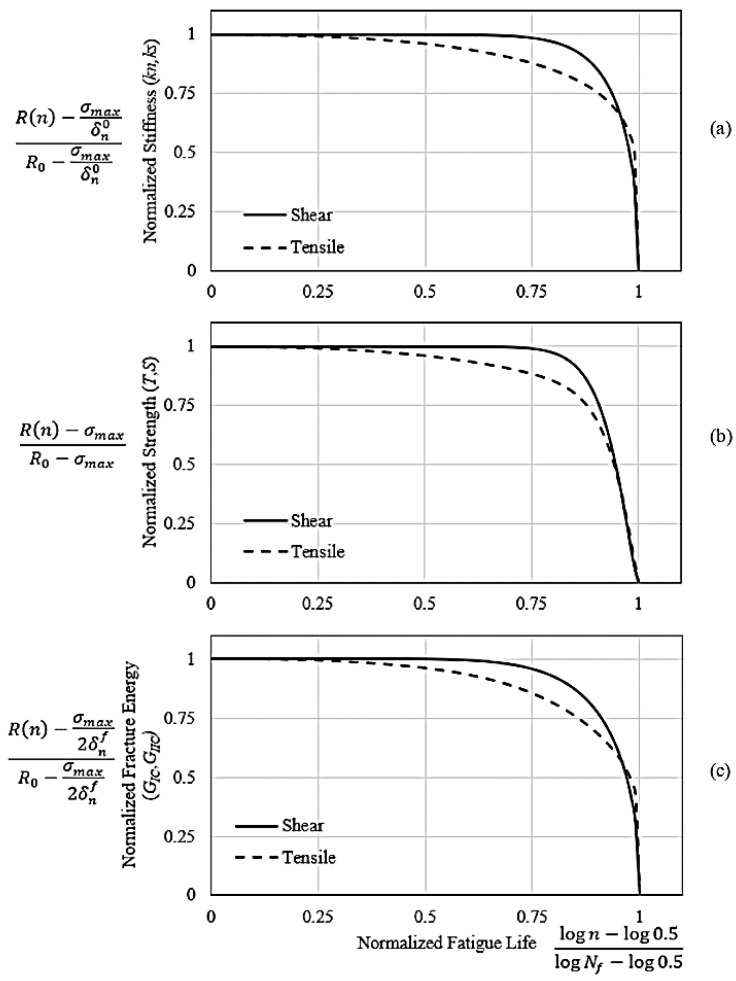
The normalized residual fatigue properties of the interface for (**a**) normal and shear penalty stiffness, (**b**) tensile and shear strength, and (**c**) fracture energy for mode I and mode II loading.

Similarly, the normalized residual fracture energy can be calculated as follows [48]:

(9) $$\frac{G_{IC}\left(n\right)-\frac{\sigma_{max}\delta_{f}^{n}}{2}}{G_{IC}^{0}-\frac{\sigma_{max}\delta_{f}^{n}}{2}}={\left\{1-{\left[\frac{\log\left(n\right)-\log\left(0.5\right)}{\log\left(N_{f}\right)-\log\left(0.5\right)}\right]}^{\mu }\right\}}^{\frac{1}{\phi }}\;$$

where $\delta_{f}^{n}$ is the relative displacement at fracture, μ, and ϕ are curve fitting parameters. The outcome of the interface normalized property model for CFRP composite laminates is displayed in Figure 3a–c. Different curve fitting parameters for the above-mentioned models are listed in Table 2. 

### 3.3. Interlaminar Fatigue Life Model

**Figure 4:**
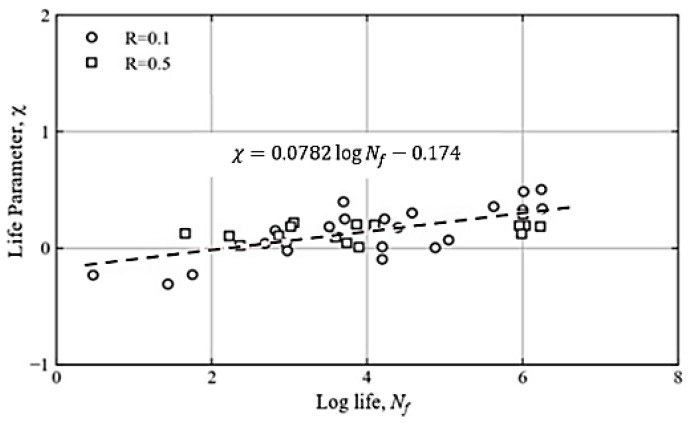
Interface fatigue life model data for CFRP composite laminates for mode I loading.

The fatigue life of an FRP composite laminate’s interface is affected by the mean stress of the loading cycle and is considered in this model through a series of fatigue life tests on FRP specimens. Double-cantilever beam (DCB) specimens were used until fracture for mode I loading [21]. Load-control-based fatigue testing at various stress levels ($\kappa ,\;\sigma_{max}$) was conducted on end-notched flexure (ENF) specimens until fracture. For mode I, identical test data for fatigue life was acquired from the published literature [34]. The life parameter *χ* is used to include the effect of mean stress in the model, as follows [3,49]:

(10) $$\chi =\log\left\{\frac{\ln\left(c\frac{\sigma_{33}^{a}}{T_{0}}\right)}{\ln\left[1-{\left(\frac{\sigma_{33}^{m}}{T_{0}}\right)}^{2}\right]}\right\}=Alog_{10}N_{f}+B$$

where *A* and *B* are curve-fitting constants. The constant *c* is optimized such that all of the data points are aligned with the best fit in a straight line when presented in the χ − log_10_ *N_f_* plot, as shown in Figure 4 with *c =* 1.939. Such a linear fit to the test data is valuable for computational life prediction exercises. By using Figure 4, the number of cycles to failure *N_f_* can be calculated for any stress ratio. 

**Figure 5:**
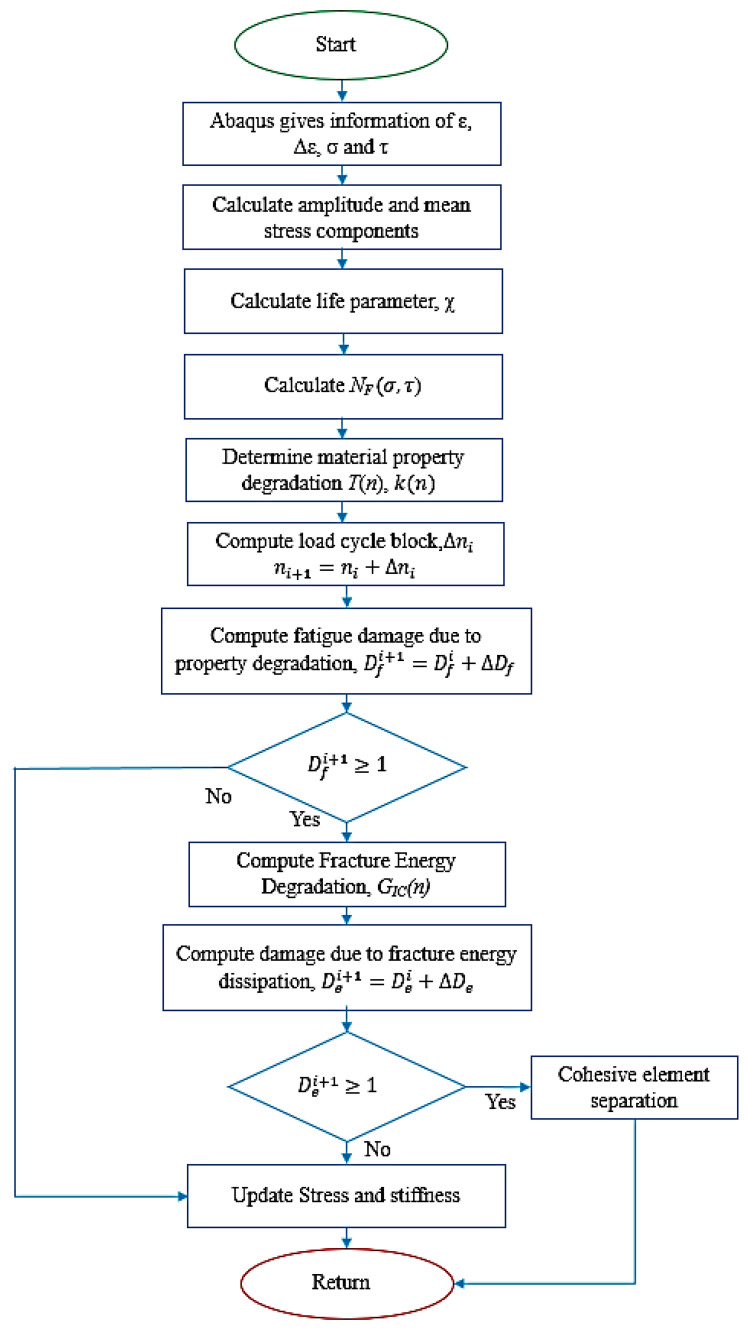
Flowchart of the interlaminar fatigue damage model for the user-defined subroutine UMAT.

The model was coded in Fortran language and integrated with the FE package [24] through a user-written subroutine (UMAT). The flowchart of the subroutine is shown in Figure 5.

## 4. Fatigue Failure Process of Mode I Interface Loading

The interface fatigue cracking process of the CFRP composite laminates under mode I loading was demonstrated through an FE simulation case study. Necessary conditions for simulating the response of a CFRP composite laminate beam with the double-cantilever beam (DCB) are discussed, including model geometry, boundary conditions, applied load cycles, and mesh convergence analysis. The results are presented here and discussed with respect to the damage evolution of the interface due to fatigue loading, the stress distribution of the interface, and the number of cycles to failure *N_f_*.

FE Simulation Case Study

**Figure 6:**
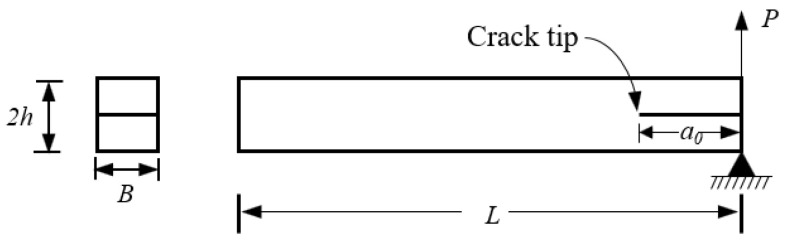
Geometry and test setup of the DCB specimen.

The DCB specimen was simulated under mode I fatigue loading conditions. The geometry and test setup of the DCB specimen are shown in Figure 6. The specimen’s nominal dimensions (in mm) were length *L* = 100, width *B* = 21, thickness *2h* = 9.6, and *a_o_* = 45. 

**Figure 7:**
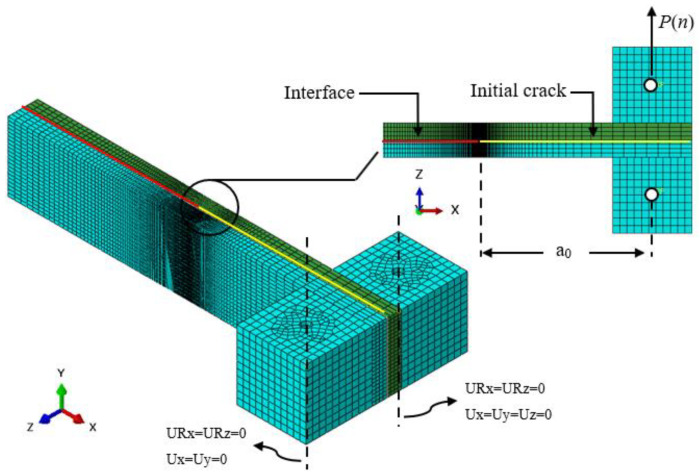
FE model of a DCB specimen demonstrating element mesh and boundary conditions.

An initial interface crack was introduced in the middle plane by overlying nodes without applying a bond between them to create a traction-free crack surface at the beginning of the simulation. The reference static material properties of the CFRP composite laminas and the critical interface of the DCB specimen employed in this simulation are shown in Table 1. The pair of loading blocks attached to the specimen were assumed to be rigid bodies. Figure 7 shows the specimen’s mesh, boundary conditions, and model geometry discretized into 3D solid elements. The lower loading block was considered non-moveable (*Ux* = *Uy* = *Uz* = 0). The load cycles were applied to the top loading block; thus, it was moveable in the z-direction (*Ux* = *Uy* = 0). Both of the loading blocks could rotate about the y-axis $\left(UR_{x}=UR_{z}=0\right)$. The load cycle blocks simulated the applied fatigue loading with *P*_max_ = 148 N and load ratio κ = 0.11. An initial loading step was defined to bring the load to the minimum level (*P*_min_ = 28 N) at the beginning of the fatigue simulation. The DCB specimen was modeled with 32 layers of laminas above the middle plane and 32 layers below the middle plane interface containing the initial crack and the cohesive layer (a total of 64 layers). The lamina was discretized into a total of 31,552 eight-node continuum shell elements (Abaqus SC8R element). This implies that 32 layers of the half-laminate are defined in 4 layers of SC8R elements. A layer of 8000 eight-node cohesive elements (Abaqus COH3D8 element) with matching nodes to the adjacent laminar surfaces is therefore prescribed.

**Figure 8:**
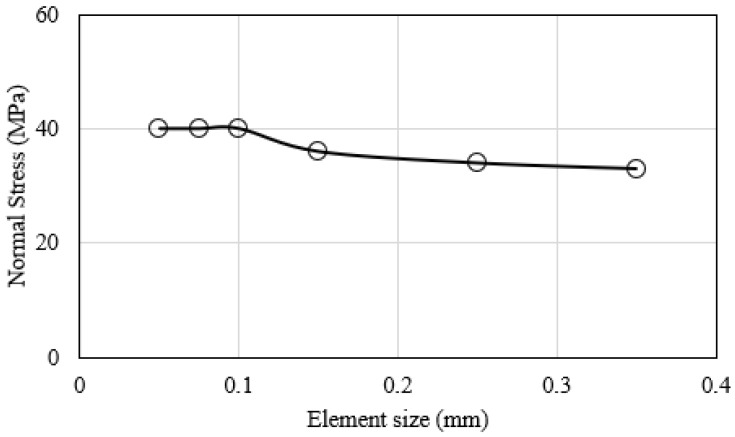
Mesh convergence analysis for the interface crack tip.

The mesh convergence analysis is shown in Figure 8. This was performed for the interface crack front region to ensure that the largest element employed had an insignificant influence on the FE-calculated variables. The interlaminar tensile stress *σ*_33_ was used as the monitoring variable in the mesh convergence analysis. In this respect, the FE model of the interface region ahead of the crack front was discretized into cohesive elements, each with an edge length of 0.1 mm along the crack growth direction.

## 5. Results and Discussion

The focus of this study was to develop a model to capture the fatigue failure process in FRP composite laminates. The results of the case study are presented and discussed in the following sections.

### 5.1. Stress Distribution of the Interface

**Figure 9:**
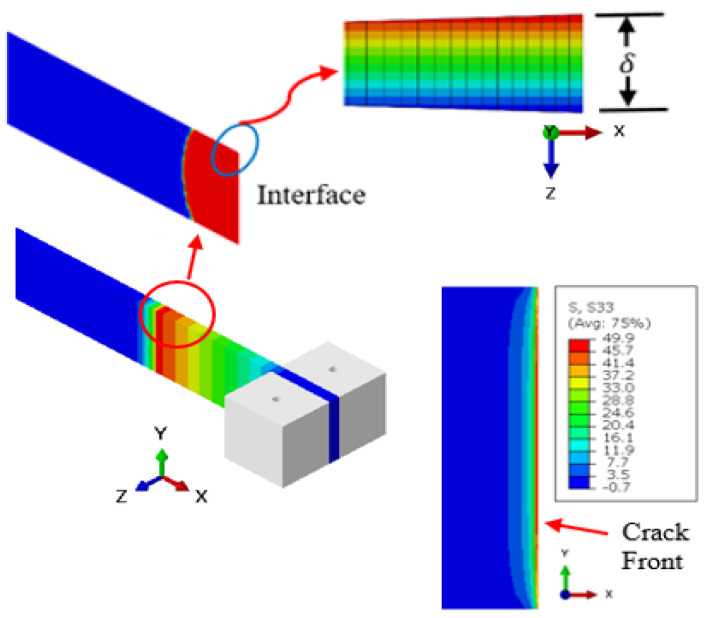
The interlaminar normal stress field in the vicinity of the starter crack tip corresponds to the peak applied load cycle at the start of the fatigue loading.

Fatigue damage accumulates due to interlaminar property degradation before the onset of interface crack nucleation. However, the stress experienced by the degrading interface material points remains unchanged. A typical interlaminar stress field corresponding to the peak applied load cycle is shown in Figure 9. The loading of the DCB beam specimen induced normal stress with a peak at 49.9 MPa. The interlaminar stress field was highly concentrated in the vicinity of the crack tip, as expected. The corresponding normal strain magnitude at the peak stress cycle was 5.12 × 10^−5^ mm. Similar stress contours but at lower magnitudes were predicted following the start of the crack nucleation process, as governed by the strain energy release rate of the interface.

**Figure 10:**
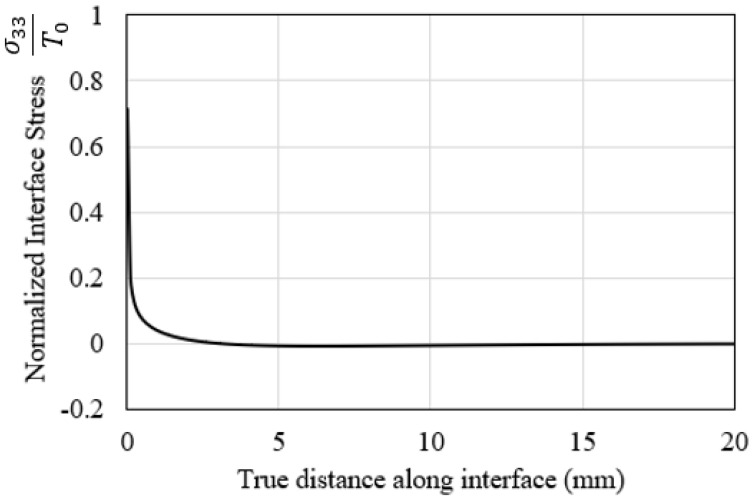
Variation of the normalized interlaminar normal stress ahead of the starter crack front at the start of the fatigue cycles.

The stress field at the crack front was analyzed further by plotting the variation in the normal stress of the interface against the true distance along the length of the interface. This effect is shown in Figure 10. The vertical axis is the stress magnitude normalized by the normal static strength of the interface. It can thus be concluded that the normal stress on the interface is highly concentrated at the crack front while sharply declining in magnitude after the length of a few elements. 

### 5.2. Evolution of the Interlaminar Fatigue Damage

**Figure 11:**
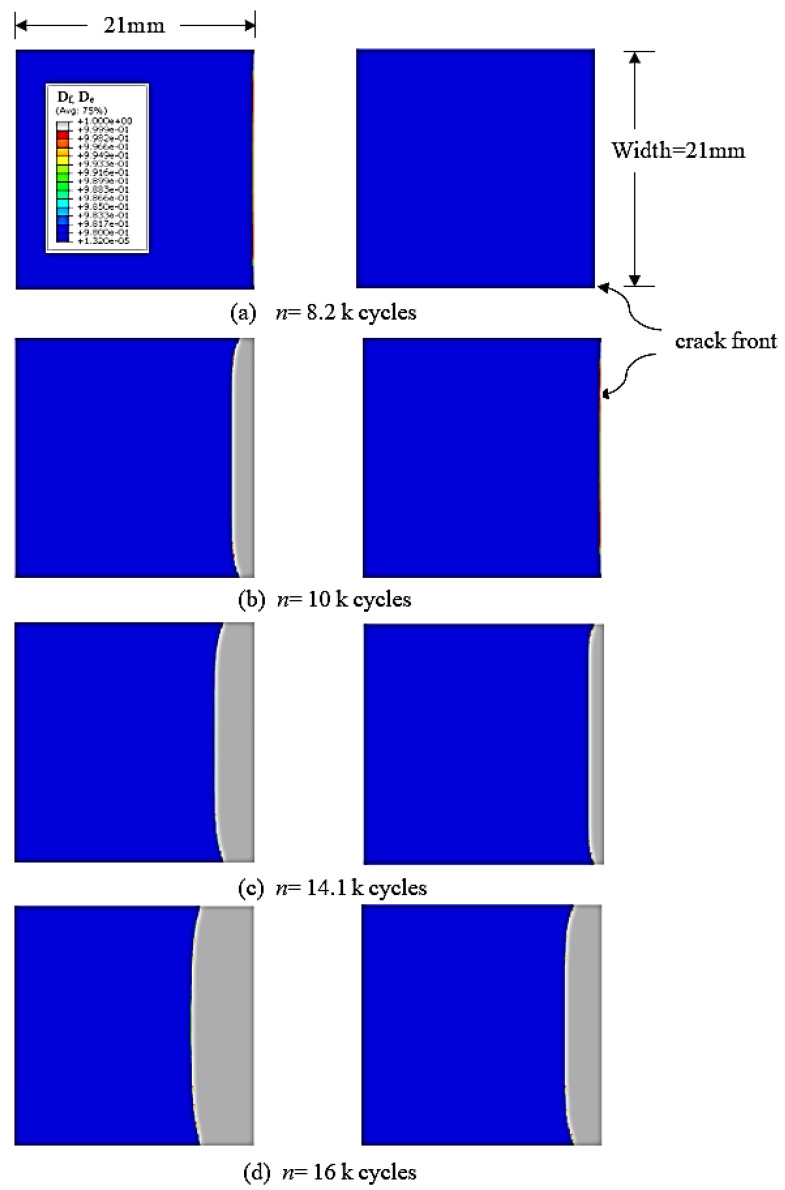
Evolution of damage variables Df (left column) and De (right column) at various stages of the fatigue cracking process: the (**a**) onset of fatigue crack nucleation, (**b**) formation of the first crack increment, (**c**) end of the interface crack growth stage with constant growth rate, and (**d**) end of the fast crack growth stage.

In this study, the interlaminar fatigue damage process was divided into two stages: A damage variable *D_f_* was used to calculate the damage until the onset of the crack nucleation in the first stage. From the onset of crack nucleation to the final separation of the material point, another damage variable *D_e_* was computed. The distribution of these damage variables is shown in Figure 11 for the different numbers of cycles. The magnitude of both variables exceeding 0.99 is assumed to satisfy the failure criteria. Figure 11a shows the first event of damage due to property degradation $D_{f}$ for the first row of elements at the crack front at almost 8200 cycles. This marks the crack nucleation onset. Figure 11b shows the separation of these damaged elements at almost 10,000 cycles, forming the first crack increment where the local stress was diminished to zero. Additional fatigue cycles forced the neighboring row of cohesive elements to separate $\left(D_{e}=1\right)$. Beyond almost 14,100 cycles, the process of separation of elements became faster, with two or more elements separating in each load block increment. Figure 11c shows the contour of damage at almost 14,100 cycles. The combination of all of these separating elements forms the fatigue crack growth stage. After this many load cycles, the crack advances at a higher rate and forms a structural interface crack. Figure 11d shows the larger interface crack increment after enduring almost 2000 more fatigue load cycles. 

**Figure 12:**
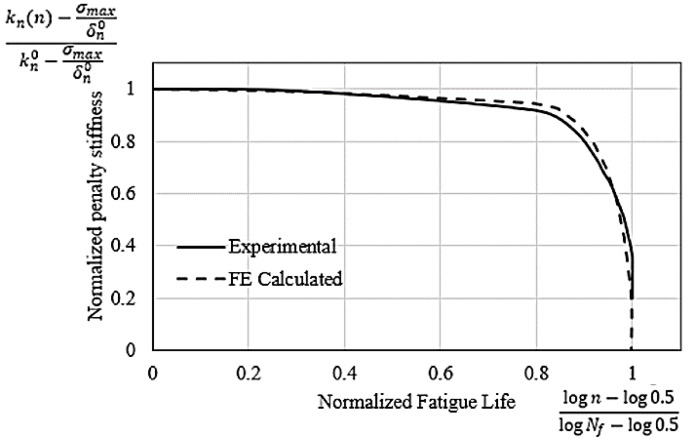
Interface normalized penalty stiffness and normalized fatigue life for experimental and FE-calculated results.

The normalized penalty stiffness and the normalized number of cycles for the experimental and FE-calculated results are compared in Figure 12. Comparing the experimental results with the FE-predicted results for the number of cycles to failure (*N_f_*) shows a very successful validation of the interlaminar fatigue damage model. 

## 6. Conclusions

In this study, a fatigue damage model was developed to predict and analyze the interface damage initiation and propagation. The fatigue model predicts the damage due to the degradation of interface properties up to the onset of fatigue crack nucleation. Fracture energy is the governing mechanism in this model for the calculation of the subsequent fatigue fracture of the interface material point. As a case study, this model was used to predict the fatigue failure behavior of CFRP laminated composite subjected to mode I fatigue load and boundary conditions. A 3D simulation of the model was carried out based on an experimentally tested CFRP DCB sample, and the FE results were compared with experimentally measured data, in which a good agreement is observed, indicating the successful validation of the model and the simulation process. The interlaminar fatigue damage model was used to illustrate the evolution of stress and quantify the fatigue crack growth in the DCB specimen. The selective results of the FE model for describing the fatigue damage evolution are as follows:

- The model successfully confined the high-stress gradient at the interface crack front region, with a normal tensile stress level of 70% of the respective interlaminar strength.
- The first event of onset of nucleation at the material point in the crack front occurred at almost 8200 cycles.
- The first increase in crack growth (i.e., the first row of elements separation) occurred at almost 10,000 cycles.
- After almost 14,000 load cycles, the crack advanced at a higher rate and formed a structural interface crack.
- A similar damage evolution process as predicted by the FE model was observed in the experimental case.
- As indicated by the results, it can be concluded that interface properties’ degradation and the dissipation of fracture energy are appropriate physical properties to be employed for interlaminar fatigue damage modeling of FRP composite laminates. Future research should aim at using a similar model for the fatigue analysis of FRP composites under mixed-mode I/II loading conditions. Another important future research direction will be to investigate the full fatigue life of FRP composite structures where fatigue damage occurs in both lamina and interface constituents, where the current interlaminar model may be integrated with a laminar fatigue damage model to predict the life of the composite structure.

## Figures and Tables

**Table 1:** Reference quasi-static lamina and interlaminar properties of CFRP composite laminates extracted based on [46,47].

	Mechanical Property	Symbol	Value
**Interlaminar**	Penalty stiffness, MPa/mm	$k_{0}^{n}$	0.974 × 10^6^
	Shear stiffness, MPa/mm	$k_{0}^{s1}$= $k_{0}^{s2}$	0.309 × 10^6^
	Tensile strength, MPa	*T*_0_	70
	Shear strength, MPa	*S*_0_	85
	Mode I critical energy release rate, N/mm	$G_{IC}^{0}$	0.31
	Mode II and III critical energy release rate, N/mm	$G_{IIC}^{0}=G_{IIIC}^{0}$	1.0
**Laminar**	Elastic modulus, GPa	*E_11_*	109
		*E_22 =_ E_33_*	8.819
	Shear modulus, GPa	*G_12 =_ G_13_*	4.315
		*G_23_*	3.2
	Poisson’s ratio	*υ _12 =_ υ_13_*	0.34
		*υ _23_*	0.38

**Table 2:** Normalized model parameters.

Residual Property	Exponent *α*	Exponent *β*	Exponent *λ*	Exponent *γ*	Exponent *Φ*	Exponent *μ*
Fracture energy, *G_IIC_*(*n*)	-	-	-	-	2.1123	8.3425
Fracture energy, *G_IC_*(*n*)	-	-	-	-	5.529	1.598
Shear stiffness, *k_s_*(*n*)	*-*	-	12.3425	2.1123	-	-
Normal stiffness, *k_n_*(*n*)	-	-	2.364	5.525	-	-
Tensile strength, *T*(*n*)	1.718	3.49	-	-	-	-
Shear strength, *S*(*n*)	0.6564	18.2375	-	-	-	-

## Data Availability

Not applicable.
